# Occupational Therapy Incorporating Cognitive Behavioral Therapy-Informed Components for Pain-Related Activity Avoidance Early After Olecranon Fracture Surgery: A Case Report

**DOI:** 10.7759/cureus.110990

**Published:** 2026-06-16

**Authors:** Naoki Ohkusa, Wataru Kukizaki, Ryota Hayasaki

**Affiliations:** 1 Department of Rehabilitation, Kenwakai Otemachi Hospital, Kitakyushu, JPN; 2 Division of Occupational Therapy, Department of Rehabilitation, Faculty of Health Sciences, Kumamoto Health Science University, Kumamoto, JPN; 3 Department of Occupational Therapy, School of Health Sciences, Sapporo Medical University, Sapporo, JPN

**Keywords:** cognitive behavioral therapy, goal setting, japanese rehabilitation, occupational therapy, postoperative pain, self-efficacy

## Abstract

Postoperative pain involves not only nociceptive components but also psychological reactions such as anxiety, pain catastrophizing, and fear-avoidance, which may delay functional recovery. Although cognitive behavioral therapy (CBT) is well established for chronic pain, evidence on the use of CBT-informed components by rehabilitation professionals in the early postoperative phase after upper-limb fracture surgery remains limited. This report describes occupational therapy (OT) incorporating CBT-informed components (hereafter, CBT-informed OT) for a patient who exhibited disproportionate pain-related fear-avoidance after olecranon fracture surgery.

A right-handed man in his 70s underwent open reduction and internal fixation with tension band wiring for a right olecranon fracture. On postoperative day three, he showed marked pain-related fear and avoidance of using the affected limb, with restricted activities of daily living (ADL) and inability to participate in work-related online meetings. Baseline assessment showed elbow active range of motion (AROM) of −45/80, pain on movement 6/10 on the Numerical Rating Scale (NRS), QuickDASH 76.5, Pain Catastrophizing Scale (PCS) 25, Pain Self-Efficacy Questionnaire (PSEQ) 20/60, and Canadian Occupational Performance Measure (COPM) performance and satisfaction scores of 2/10 for all identified goals. Because the baseline PCS was below the conventional cutoff, the clinical formulation identified low pain self-efficacy and fear-avoidance, rather than catastrophizing alone, as the central psychological drivers. A 12-week CBT-informed OT program was provided in three phases: (1) psychoeducation and cognitive restructuring, (2) graded goal-directed activity guided by COPM and the Aid for Decision-making in Occupation Choice for Hand (ADOC-H), and (3) pacing and self-management for return to occupational roles.

At 12 weeks, the patient showed marked improvements: AROM −10/130, pain on movement 2/10 (change: −4), QuickDASH 12.5 (change: −64.0), PCS 1 (change: −24), and PSEQ 58 (change: +38). COPM performance and satisfaction improved from 2 to 10 (change: +8) for both short-term goals (independence in dressing and bathing) and the long-term goal (participation in work-related online meetings). The QuickDASH Work subscale, not assessed at baseline due to hospitalization, was 25 at 12 weeks, corresponding to mild-to-moderate residual difficulty.

This single case suggests that CBT-informed OT may be a feasible approach for addressing pain-related fear-avoidance in the early postoperative phase after upper-limb fracture surgery. The temporally associated improvements in self-efficacy and occupational performance are consistent with the clinical formulation, although natural postoperative recovery cannot be excluded as a contributing factor. Controlled studies are needed to evaluate efficacy.

## Introduction

Postoperative pain is not merely physical discomfort but is accompanied by psychological reactions such as anxiety and pain catastrophizing, which can hinder physical activity and social reintegration [1]. In particular, fear-avoidance beliefs related to pain have been reported to delay functional recovery and contribute to the development of chronic pain [2,3]. Inadequate management of these psychological factors remains a global challenge in pain rehabilitation, as it is a major risk factor for the transition from acute to chronic pain [2,3].

Cognitive behavioral therapy (CBT) is a psychological intervention that focuses on the interactions between pain-related cognitions, emotions, and behaviors. It promotes behavioral change by modifying maladaptive thought patterns and enhancing self-efficacy. The efficacy of formal CBT for chronic pain, typically delivered by clinical psychologists or other mental health professionals as a structured psychotherapy, is widely recognized [4]. In rehabilitation settings, however, the core components of CBT are often not delivered as a stand-alone psychotherapy but are incorporated into routine therapy by non-psychologist clinicians as CBT-informed strategies, including psychoeducation, cognitive restructuring, graded goal setting, pacing, and self-management. The present report describes this latter approach, which we refer to throughout as occupational therapy (OT) incorporating CBT components, or CBT-informed OT. We use this terminology deliberately to indicate that the intervention was delivered within the professional scope of OT and that it did not constitute a full course of formal CBT.

Upper limb fractures, particularly those around the elbow joint, pose specific clinical challenges due to their anatomical characteristics, often leading to early postoperative contractures. Because the elbow tolerates immobilization poorly, even short periods of guarded movement can result in stiffness that subsequently interferes with dressing, bathing, desk-based work, and other daily activities that depend on coordinated reaching, lifting, and forearm rotation. Avoidance of using the affected limb due to pain or anxiety may initially present as protective non-use, voluntary guarding driven primarily by pain-related fear in the acute phase, which, if sustained, may evolve into a more persistent fear-related disuse pattern in which the limb is excluded from spontaneous activity despite recovered capacity, potentially worsening functional recovery. Therefore, there is a high demand for psychological approaches that break the vicious cycle of fear-avoidance behavior and encourage early active use, rather than relying solely on conventional exercise therapy [2,3]. Within an OT framework, such psychological approaches can be naturally embedded in occupation-based practice, in which meaningful daily activities themselves serve as the medium for modifying fear-related cognitions, building self-efficacy, and promoting behavioral change.

Despite this need, comprehensive reports on the use of CBT-informed components by rehabilitation professionals for patients with acute postoperative pain remain scarce [5]. Regarding occupational therapy (OT) focusing on psychological aspects for early postoperative pain, Hara et al. [5] reported an OT practice using CBT components after high tibial osteotomy (HTO). However, that study focused on lower limb and gait function; to our knowledge, there is limited published evidence on OT-led, CBT-informed care that specifically targets pain-related fear avoidance in the early postoperative phase after upper-limb fracture surgery, where the rehabilitation focus shifts to fine upper-limb function for self-care and vocational activities.

In this case report, we describe a CBT-informed OT intervention, incorporating cognitive restructuring, goal setting, and pacing as CBT components delivered within an OT framework, for a patient with an olecranon fracture. This patient initially avoided using the affected limb due to early postoperative pain and psychological factors, leading to marked impairments in activities of daily living (ADLs) and vocational tasks. Because the evidence base in this specific population is limited, this report is intended to describe the clinical reasoning, intervention structure, feasibility, and observed patient course of CBT-informed OT for early postoperative pain, rather than to test the efficacy of formal CBT.

## Case presentation

Patient information and clinical findings

**Figure 1:**
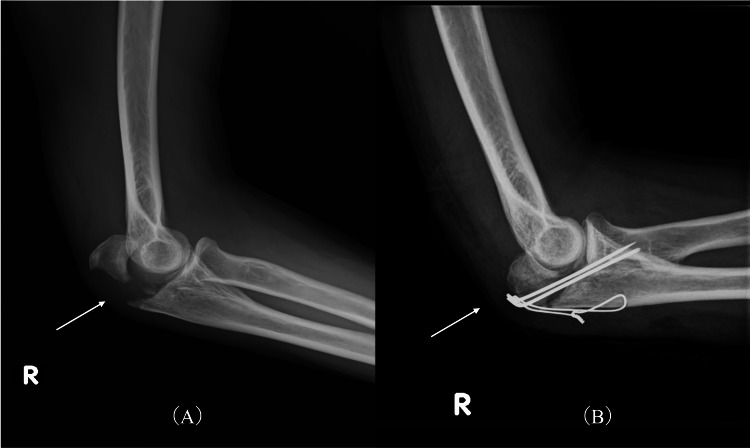
Preoperative and postoperative lateral radiographs of the right elbow (A) Preoperative lateral radiograph showing an olecranon fracture. (B) Postoperative lateral radiograph after open reduction and internal fixation with tension band wiring, showing improved alignment. The arrow in panel A indicates the olecranon fracture line. The arrows in panel B indicate the Kirschner wires and the tension band of the tension band wiring construct used for open reduction and internal fixation. The postoperative radiograph confirms anatomical reduction of the fracture and stable fixation.

The patient was a right-handed man in his 70s who lived with his wife. He sustained a right olecranon fracture after a fall and subsequently underwent open reduction and internal fixation (ORIF) using the tension band wiring technique. Preoperative and postoperative radiographs of the right elbow are shown in Figure 1. His occupation was primarily desk-based and required frequent participation in online meetings. He had no significant past medical history, including psychiatric disorders.

Postoperative management and early progress

Following surgery, the patient's right elbow was immobilized with a splint for one week. Although splint removal was permitted during OT sessions, he was instructed to remain non-weight-bearing on the affected limb until bone union was confirmed. For pain management, acetaminophen-based analgesics were prescribed for four weeks postoperatively. From the early postoperative phase, the patient reported intense pain and expressed marked anxiety regarding his ADL and work-related activity.

Pre-intervention clinical progress

Physical therapy was initiated on the first postoperative day, focusing on range-of-motion exercises for adjacent joints, specifically the shoulder and wrist, and instruction on limb elevation to manage edema. By the third postoperative day, when the initial OT assessment was conducted, the patient exhibited intense fear and anxiety related to pain. He expressed his concerns verbally, stating, "I'm afraid the pain will get worse," and "Won't it get worse if I move it too much?" During rehabilitation sessions, his sitting posture at the desk remained unstable due to a fear of contact pain at the olecranon, leading to a persistent tendency to avoid using the affected upper limb. Distinguishing this presentation from expected postoperative caution. While some pain-related guarding is expected after olecranon ORIF, the patient's response was considered disproportionate to the surgical precautions in three respects: (1) his avoidance extended to movements explicitly permitted by the surgical team (e.g., active elbow motion within the pain-free range after splint removal); (2) the avoidance persisted despite verbal reassurance and observation that movement did not worsen objective findings; and (3) it prevented participation in safe rehabilitation tasks and basic self-care that would otherwise have been feasible given his physical status. Since these psychological factors, combined with acute pain, hindered his active participation in functional training, we determined that an OT intervention specifically addressing these psychological aspects was essential.

Initial assessment

OT assessment was conducted on the third postoperative day (Table 1). The presenting features that led us to identify this case as pain-related activity avoidance-spanning pain intensity, maladaptive beliefs, avoidance behaviors, functional limitations, and psychological scores-are summarized as a recognition profile for clinicians evaluating similar postoperative patients (Table 2).

**Table 1:** Changes in clinical, pain-related, and occupational outcome measures from postoperative day 3 to postoperative week 12 PROM: passive range of motion; AROM: active range of motion; NRS: Numerical Rating Scale; QuickDASH: Quick Disabilities of the Arm, Shoulder, and Hand; PCS: Pain Catastrophizing Scale; PSEQ: Pain Self-Efficacy Questionnaire; COPM: Canadian Occupational Performance Measure. Negative values for elbow extension indicate lack of full extension. Higher QuickDASH and PCS scores indicate greater upper-limb disability and greater pain catastrophizing, respectively; higher PSEQ scores indicate greater pain self-efficacy; higher COPM performance and satisfaction scores indicate better perceived occupational performance and greater satisfaction. POD: postoperative day; PROM: passive range of motion; AROM: active range of motion; NRS: Numerical Rating Scale; PCS: Pain Catastrophizing Scale; PSEQ: Pain Self-Efficacy Questionnaire; QuickDASH: Quick Disabilities of the Arm, Shoulder and Hand; COPM: Canadian Occupational Performance Measure.

Outcome measure	Initial evaluation (POD 3)	Final evaluation (postoperative week 12)
Elbow PROM, extension/flexion (°)	–40/100	–5/140
Elbow AROM, extension/flexion (°)	–45/80	–10/130
Pain at rest, NRS (0–10)	4	1
Pain during movement, NRS (0–10)	6	2
PCS (0–52)	25	1
PSEQ (0–60)	20	58
QuickDASH Disability/Symptom score (0–100)	76.5	12.5
QuickDASH Work module (0–100)	Not assessed	25
COPM mean performance score (1–10)	2	10
COPM mean satisfaction score (1–10)	2	10

**Table 2:** Recognition profile of pain-related activity avoidance at postoperative day three This table summarizes the presenting features at postoperative day three that led the clinical team to identify this case as pain-related activity avoidance, presented as a recognition profile for clinicians evaluating similar postoperative patients. NRS: Numerical Rating Scale (0–10; higher scores indicate greater pain intensity); PCS: Pain Catastrophizing Scale (0–52; higher scores indicate greater pain catastrophizing; commonly cited clinical cutoff is 30); PSEQ: Pain Self-Efficacy Questionnaire (0–60; higher scores indicate greater pain self-efficacy); QuickDASH: Quick Disabilities of the Arm, Shoulder, and Hand (0–100; higher scores indicate greater upper-limb disability); COPM: Canadian Occupational Performance Measure (1–10; higher scores indicate better perceived occupational performance and greater satisfaction); POD: postoperative day; ORIF: open reduction and internal fixation. The criteria used to distinguish this presentation from expected postoperative caution are detailed in the Pre-intervention Clinical Progress subsection (Distinguishing this presentation from expected postoperative caution).

Domain	Findings in the present case	Clinical descriptor
Pain intensity	NRS 4 at rest; NRS 6 during movement	Moderate resting pain disproportionate to expected POD 3 trajectory, with substantial movement-evoked pain
Maladaptive beliefs	"I'm afraid the pain will get worse." "Won't it get worse if I move it too much?" "It's not getting better at all despite the surgery."	Catastrophic interpretation of pain; expectation that movement causes further harm; pessimistic appraisal of recovery
Avoidance behaviors	Limb pulled toward trunk; unstable sitting posture due to fear of contact pain at olecranon; avoidance of movements explicitly permitted by the surgical team	Protective non-use disproportionate to surgical precautions; persists despite reassurance
Functional limitations	Unable to dress or bathe independently; unable to engage in desk-based work or online meetings	Restricted participation in basic self-care and vocational roles that would otherwise be feasible given the patient's physical status
Psychological scores	PCS 25/52 (below clinical cutoff of 30); PSEQ 20/60 (markedly low); QuickDASH 76.5 (substantial disability); COPM performance and satisfaction 2/10	Low pain self-efficacy and pain-related fear avoidance as central drivers; catastrophizing present but not severe

Physical Function

The passive range of motion (PROM) for the elbow was −40° in extension and 100° in flexion, while the active range of motion (AROM) was −45° in extension and 80° in flexion. Pain intensity, measured by the Numerical Rating Scale (NRS), was four at rest and six during motion. No neurological impairments, such as ulnar nerve palsy, were observed. Upper limb function was evaluated using the Quick Disabilities of the Arm, Shoulder, and Hand (QuickDASH) questionnaire, with a score of 76.5, indicating substantial disability [6]. Vocational function was not assessed at this time as the patient was still hospitalized.

Psychological Status

The Pain Catastrophizing Scale (PCS) score was 25/52 [7]. Although this value was below the commonly cited clinical cutoff of 30 [8], the score reflected substantial pain-related rumination and helplessness within an individual context. The Pain Self-Efficacy Questionnaire (PSEQ) score was 20/60, indicating markedly low self-efficacy for performing activities despite pain [9]. Considered together, the psychological profile in this case suggested that low pain self-efficacy and fear-related activity avoidance, rather than catastrophizing alone, were the central psychological drivers of disability, a formulation that subsequently guided the intervention plan.

Interview and Goal Setting

An evaluation interview using the Canadian Occupational Performance Measure (COPM) [10] was conducted to identify client-centered needs and set goals. To complement this with concrete identification of upper-limb-related activities, the Aid for Decision-making in Occupation Choice for Hand (ADOC-H) [11] was utilized. These two tools served complementary roles: ADOC-H, an illustrated decision-making aid, was used to elicit and specify concrete hand- and upper-limb-related activities relevant to the patient's daily life, while COPM was used to rate the importance, performance, and satisfaction of the selected occupational goals and to track their change over time. The patient identified "independence in dressing and bathing" as one short-term occupational goal and "participation in work-related online meetings" as one long-term occupational goal. All goals were assigned an importance rating of 10/10. However, both performance and satisfaction scores were 2/10 (Table 3).

**Table 3:** COPM-identified occupational goals and ratings at initial and final evaluation COPM: Canadian Occupational Performance Measure. Importance, performance, and satisfaction are rated on a 10-point scale. Although dressing and bathing are functionally distinct activities, the patient elected to track them as a single paired self-care domain on the COPM because they shared overlapping upper limb demands (one-handed sleeve passage, buttoning, overhead reach, and reaching across the body) and were performed within the same morning self-care routine. Separate functional outcomes for dressing and for bathing are reported in the Outcomes section of the main text. COPM: Canadian Occupational Performance Measure; POD: postoperative day.

COPM-identified occupational goal	Time frame	Importance (1–10)	Performance (POD 3)	Performance (week 12)	Satisfaction (POD 3)	Satisfaction (week 12)
Independence in dressing and bathing	Short-term	10	2	10	2	10
Participation in work-related online meetings	Long-term	10	2	10	2	10

During the interview, the patient remarked, "The pain is so strong that I can't use my arm. I can't even get dressed or take a bath by myself," and "It would be a problem if I couldn't attend online meetings for work." These statements were consistent with his low PSEQ score. Furthermore, his comment, "It's not getting better at all despite the surgery," indicated a discrepancy in his perception of the postoperative recovery process and a catastrophic interpretation of pain.

Case conceptualization

**Figure 2:**
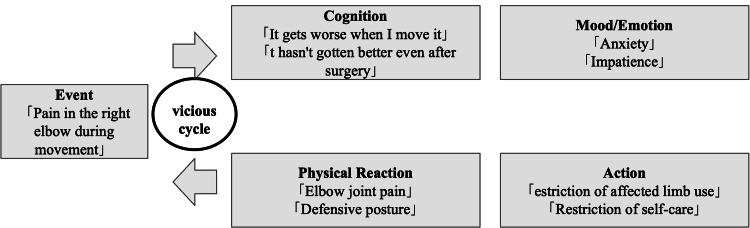
Baseline case conceptualization of pain-related fear and avoidance on postoperative day three (POD 3) Acute postoperative pain triggered anxiety, frustration, and threat-based interpretations of movement. These responses were associated with guarding, avoidance of affected upper-limb use, unstable desk sitting because of fear of contact pain at the olecranon and reduced occupational performance in self-care activities. The figure summarizes the hypothesized cycle linking pain, fear, avoidance, and reduced participation in dressing, bathing, and desk-based work-related activity at the initial OT evaluation. Bold arrows indicate hypothesized reinforcing pathways within the cycle; the closing arrow returning from the "Consequence" node to the "Pain" node is labeled "vicious cycle" to emphasize the self-reinforcing nature of the pathway. This figure represents a clinical case conceptualization based on the fear-avoidance model [12] and is not intended to demonstrate a proven causal mechanism in this single case. POD: postoperative day; OT: occupational therapy.

Figure 2 illustrates the baseline case conceptualization.

The patient's acute pain triggered anxiety and frustration, which, combined with low confidence in his ability to act despite pain, drove a guarding posture and active avoidance of upper-limb use. Behaviorally, this led to a contraction of his activity range. Although the patient was compliant with prescribed medications and attempted the instructed exercises, the resulting pain on movement further reinforced his belief that movement was harmful, perpetuating a vicious cycle of fear avoidance and low self-efficacy.

OT intervention plan

A CBT-informed OT intervention was designed to facilitate the achievement of goals identified through COPM. Based on the patient's statements, such as "It's not getting better at all despite the surgery" and "I get anxious whenever I feel pain," we determined that his current psychological and cognitive state made spontaneous problem-solving difficult. Therefore, immediate implementation of interventions solely focused on behavior change was deemed premature. Although the patient's PCS score was below the clinical cutoff of 30 points, according to the fear-avoidance model proposed by Vlaeyen et al., a CBT-informed approach is indicated whenever pain-related fear and avoidance behaviors are observed, regardless of the total PCS score [12]. During initial training sessions, we observed a guarding posture, characterized by pulling the affected limb toward the trunk, and an unstable sitting posture due to fear of contact pain at the olecranon. These clinical observations were consistent with the "avoidance" component of the fear-avoidance model, justifying the need for a psychological approach using CBT-informed techniques. Given the psychological profile (PCS below cutoff but markedly low PSEQ), the intervention was framed to prioritize self-efficacy building and fear-avoidance reduction, with catastrophizing addressed as a secondary target through psychoeducation.

The intervention was structured into three phases

Phase 1: Cognitive Restructuring and Psychoeducation

Priority was given to cognitive restructuring to improve the patient's psychological state. Through psychoeducation, we aimed to help the patient manage his anxiety and gain a broader perspective on his recovery process.

Phase 2: Goal Setting and Gradual Activity Resumption

Once the patient was able to manage his anxiety and analyze his situation objectively, the intervention moved to the second phase. This phase focused on setting specific goals using the COPM and ADOC-H and promoting the acquisition of self-management skills through the gradual resumption of daily activities.

Phase 3: Pacing and Behavioral Change

The final phase involved behavioral change through pacing. The objective was to accumulate successful experiences through the actual performance of ADLs (dressing and bathing) and instrumental ADLs (PC operation and participation in online meetings). After each session, an interview based on a structured agenda (e.g., reflecting on tasks, setting today's goals, and establishing the next session's challenges) was conducted. This allowed for continuous reflection, breaking down goals into achievable steps, and iterative evaluation to refine the OT program.

Therapeutic process

The patient was discharged two weeks postoperatively and received outpatient rehabilitation until the 12th postoperative week. The intervention frequency was 40 minutes per session, conducted five times a week during the inpatient phase (postoperative day 3 to week 2) and once a week during the outpatient phase (weeks 3 to 12). Adherence to scheduled sessions was 100%, and the patient reported completing the agreed-upon home practice, including limb elevation, prescribed range-of-motion exercises, and graded daily activities, on most days. No adverse events such as wound complications, hardware-related problems, or pain exacerbations requiring medical intervention occurred during the intervention period. The therapeutic process was divided into three distinct phases.

Phase 1: Cognitive Restructuring (Postoperative Day 3 to Week 2)

To facilitate behavioral change, we focused on identifying and correcting cognitive distortions. Three individual psychoeducation sessions were conducted to encourage adaptive thinking. Using a pamphlet developed based on previous research [13], we explained: (1) the physiological mechanisms of pain, (2) the discrepancy between pain intensity and tissue damage, and (3) the risk that excessive rest and avoidance behavior could lead to decreased activity and prolonged pain [14]. We shared the principle of "activity within tolerable pain" rather than "total pain avoidance." To address the patient's belief that "movement will make the condition worse," the therapist used explanations such as "After surgery, your tissues are healing, and pain is a normal signal during this process. But pain does not always mean that you are damaging the tissue. Gentle movement, within a range that does not progressively worsen your pain, actually helps the healing tissues regain their flexibility. If we avoid movement completely, your elbow may become stiff, which would make daily activities harder than the pain itself." This explanation was paired with in-session demonstration: the therapist guided the patient through a small, controlled elbow movement; monitored NRS before, during, and 30 minutes after; and showed him that pain returned to baseline without worsening, providing experiential counter-evidence to the harm belief. However, because the patient's baseline resting pain was already NRS 4, we did not rely on a rigid numerical cutoff alone. Instead, we used an individualized pain-monitoring approach in which activity was continued when pain (i) remained tolerable to the patient, (ii) did not progressively worsen during the task, (iii) returned to baseline within approximately 30 minutes after the session, and (iv) was not accompanied by warning signs (e.g., new swelling, redness, or paresthesia). This approach was informed by postoperative pain management guidance and by the concept of safe movement that does not imply further tissue injury [12]. Initially, the patient expressed anxiety and frustration, stating, "It will get worse if I move it" and "It will never return to normal." However, after reviewing his daily abilities (e.g., "I can move it once the splint is removed"), his anxiety decreased. He eventually remarked, "It's okay to move even if it hurts" and "I should just take it step by step." Based on these adaptive thoughts, we proceeded to Phase 2.

*Phase 2: Goal Setting and Self-Management (Weeks 2 to 4)* 

Goals identified through COPM and ADOC-H were broken down into achievable sub-goals to facilitate a gradual return to activities. For dressing, we practiced a graded approach: passing the sleeve through the affected limb first, followed by buttoning, and finally overhead movements. For bathing, the use of assistive devices based on the reach of the affected limb was considered. Regarding participation in work-related online meetings, specific guidance was provided on ergonomic PC posture, upper-limb support on the desk, monitor, and camera positioning to reduce reaching demand, and environmental adjustments such as forearm padding to mitigate olecranon contact pain. During each session, NRS and anxiety levels were monitored so that feedback could be provided when the patient performed tasks within a safe and tolerable range. At the week four reassessments, pain on movement had decreased to NRS 3, AROM had improved to approximately −25/110, PCS to 10, and PSEQ to 38; the patient had become independent in dressing and was performing bathing with minimal assistance. We reinforced the concepts that pain is not always synonymous with tissue damage, and that avoidance could lead to functional decline [14]. The patient stated, "The pain is still there, but it's better than before. I think I can manage it better now," indicating positive progress in pain management.

*Phase 3: Behavioral Change Through Pacing (Weeks 4 to 12)* 

After the patient acquired self-management skills, pacing was implemented to promote work-related activities (Table 4). Two additional psychoeducation sessions were conducted to reinforce previous learning and introduce activity regulation (e.g., scheduled rest, gradual load increase) and coping strategies for pain exacerbation. ADLs (dressing, bathing, and stair climbing) and instrumental ADLs (cooking, laundry, and cleaning) were practiced incrementally between weeks four and five. For vocational return at week eight, we supported ergonomic adjustments and time management (e.g., inserting rest breaks every 25-30 minutes of desk work). Pacing decision criteria. Progression to a higher activity level (longer task duration, greater load, or more complex task) was made when the following criteria were jointly met across at least two consecutive sessions: (a) NRS during the task ≤ 3, (b) self-reported anxiety remained low or absent, (c) pain returned to baseline within 30 minutes after the activity, (d) the task could be completed without compensatory posture or guarding, and (e) no warning signs were observed. When these criteria were not met, the activity was held at the current level or temporarily downgraded, and rest breaks were extended. At week eight, the patient had resumed full 30-minute online meetings with one scheduled mid-meeting micro-break and used forearm padding for olecranon support; AROM had reached approximately −15/120, NRS on movement was two, and PSEQ was 50. Through pacing, the patient progressively increased his activity level without exacerbating his pain. He demonstrated a proactive attitude, stating, "I'll do things at my own pace." The OT intervention was concluded after the final assessment confirmed successful behavioral adaptation and improved pain management.

**Table 4:** Representative progression of activity resumption during Phase 3 (postoperative weeks 4 to 12) ADL: activities of daily living; IADL: instrumental activities of daily living; NRS: Numerical Rating Scale. Progression to a higher activity level (longer task duration, greater load, or more complex task) was made when the following criteria were jointly met across at least two consecutive sessions: (a) NRS during the task ≤ 3, (b) self-reported anxiety remained low or absent, (c) pain returned to baseline within 30 minutes after the activity, (d) the task could be completed without compensatory posture or guarding, and (e) no warning signs were observed. When these criteria were not met, the activity was held at the current level or temporarily downgraded, and rest breaks were extended. ADL: activities of daily living; PC: personal computer.

Time point	Target activities	Pacing strategy and clinical focus	Progression criteria applied at this stage
Postoperative week 4	Basic self-care activities, including dressing and bathing	Practice focused on short task bouts, upper-limb support, and symptom monitoring.	NRS during task ≤ 3; pain returned to baseline within 30 min; no compensatory posture; no warning signs (across ≥2 consecutive sessions)
Postoperative week 5	Instrumental ADL tasks and desk-based activity	Cooking, laundry, and cleaning were added. PC work was practiced for 15 minutes followed by a planned rest break.	NRS during task ≤ 3; low/absent anxiety; pain returned to baseline within 30 min; rest breaks every 20–30 min as needed
Postoperative week 8	Increased desk-based activity and preparation for work-related online meetings	PC work was practiced for 30 minutes followed by a planned rest break. Mock practice for work-related online meetings was introduced.	NRS during task ≤ 3; low/absent anxiety; ergonomic adjustments (forearm padding) maintained; no warning signs
Postoperative week 12	Participation in work-related online meetings and broader daily activities	The patient participated in work-related online meetings using self-managed pacing, scheduled rest breaks, and environmental adjustment as needed.	All five progression criteria met across multiple sessions; intervention concluded

Outcomes

Final Assessment at 12 Weeks Postoperatively

At the final assessment conducted 12 weeks postoperatively, the patient demonstrated marked improvements in range of motion (ROM), pain intensity, psychological indices, and occupational performance (Table 1). Interpretive direction for the scales used is as follows: higher QuickDASH and PCS scores indicate greater disability and greater catastrophizing, respectively; higher PSEQ and COPM scores indicate greater pain self-efficacy and better occupational performance and satisfaction, respectively.

Physical Function and Pain

Passive ROM improved from −40/100 to −5/140 (change: +35 extension, +40 flexion), and active ROM improved from −45/80 to −10/130 (change: +35 extension, +50 flexion). Pain intensity (NRS) decreased from 4 to 1 at rest (change: −3) and from 6 to 2 during motion (change: −4). The QuickDASH disability/symptom score improved from 76.5 to 12.5 (change: −64.0). The QuickDASH "Work" subscale was not assessed at baseline because the patient was hospitalized and not yet engaged in vocational tasks; this measure was therefore obtained only at the 12-week mark, when it was 25. A QuickDASH Work score of 25 corresponds to mild-to-moderate residual difficulty with work-related upper-limb tasks; in this patient, it was consistent with his self-report of independently performing desk work and online meetings while still requiring scheduled rest breaks and ergonomic adjustments, including forearm padding for olecranon support.

Psychological Status

The PCS score decreased from 25 to 1 (change: −24), and the PSEQ score increased from 20 to 58 (change: +38). Given that the baseline PCS was below the conventional cutoff of 30, the magnitude of PCS reduction should be interpreted within the context of an already non-severe catastrophizing profile; in contrast, the change in PSEQ moved the patient from markedly low to near-ceiling self-efficacy, consistent with the case formulation that low self-efficacy was a central psychological driver in this case.

Occupational Performance

**Figure 3:**
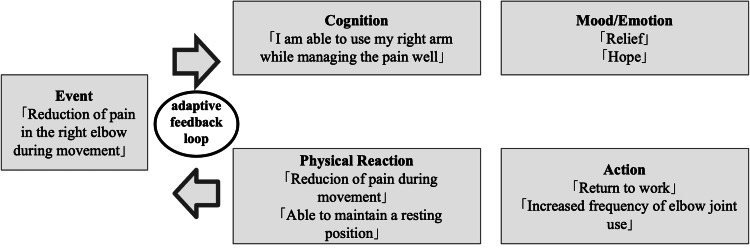
Updated case conceptualization after occupational therapy incorporating CBT components at postoperative week 12 After OT incorporating CBT components, the patient showed more adaptive pain-related beliefs, improved self-efficacy, and greater tolerance of graded activity. These changes were associated with reduced avoidance of the affected upper limb, improved performance in dressing and bathing, and participation in work-related online meetings using pacing and environmental adjustment. The figure summarizes the hypothesized changes in cognition, behavior, and occupational performance at the final evaluation. Bold arrows indicate the adaptive pathways within the cycle; the closing arrow returning from the "Consequence" node is labeled "adaptive feedback loop" to provide a direct visual contrast with the "vicious cycle" of Figure 2. This figure represents a clinical case conceptualization and is not intended to demonstrate a proven causal mechanism in this single case. OT: occupational therapy; CBT: cognitive behavioral therapy.

The evaluation using the Canadian Occupational Performance Measure (COPM) showed that both identified occupational goals, the short-term goal of independence in dressing and bathing, and the long-term goal of participation in work-related online meetings, were achieved. Performance and satisfaction scores improved from 2 to 10 for all goals (change: +8) (Table 3). Concretely, for dressing, the patient regained independence in upper-body dressing, including sleeve passage through the affected limb, buttoning, and overhead movements, with no compensatory strategies remaining necessary. For bathing, the patient regained independence in washing his back and reaching toward overhead shower controls; he continued to use a long-handled sponge as a comfort measure but no longer required assistance. For online meetings, the patient resumed full participation in scheduled work meetings, maintaining seated desk posture for 30 to 60 minutes, operating keyboard and mouse bilaterally, and using forearm padding for olecranon contact comfort; the only residual strategy was scheduled micro-breaks every 25 to 30 minutes. Although the COPM performance and satisfaction scores for participation in work-related online meetings reached 10, the QuickDASH Work module score remained 25 at week 12. This discrepancy may be explained by differences in the constructs measured by these tools: the COPM reflected achievement of the patient-specific goal of participating in online meetings, whereas the QuickDASH Work module captured broader upper-limb difficulties during work-related tasks. Figure 3 shows the updated case conceptualization after occupational therapy incorporating CBT components at postoperative week 12.

## Discussion

The most important finding of this case report is that OT incorporating CBT components was feasible for a patient with intense early postoperative pain and pain-related activity avoidance after olecranon fracture surgery and that it was temporally associated with improvement in psychological and occupational outcomes. For patients suffering from severe pain and distress, implementing behavioral change is inherently challenging. Therefore, we prioritized the identification and restructuring of maladaptive cognitions to facilitate a more accurate perception of reality and alleviate psychological symptoms. Previous literature suggests that graded goal setting and monitoring of pain and activity may support pain management and activity resumption after orthopedic surgery [13]. While Hara et al. [5] reported the usefulness of OT incorporating CBT components in a lower-limb case after HTO, the present report complements that work by demonstrating the applicability of a similar approach to upper-limb fracture rehabilitation. In this case, psychoeducation was utilized as a primary tool for cognitive restructuring, providing information on pain physiology and the discrepancy between pain intensity and actual tissue damage [14]. By addressing the patient’s belief that "movement worsens the condition" with the counter-evidence that "pain is not always synonymous with tissue damage" and "movement within a safe range facilitates recovery," and by repeatedly confirming during sessions that the patient’s activity level remained within a tolerable range, we helped transform theoretical understanding into practical confidence. The substantial reduction in his PCS score from 25 to 1 likely reflects this cognitive shift. Riddle et al. reported that goal setting is essential for the effective self-management of pain and anxiety following cognitive restructuring. They noted that coping skills, such as temporary pain management strategies, self-relaxation, and activity management, improve psychological well-being and functional capacity in patients with musculoskeletal disorders [15]. In our intervention, we utilized COPM and ADOC-H to clarify goals directly linked to the patient's life roles and broke these down into achievable sub-goals to foster a series of successful experiences. This process likely contributed to the improvement in his self-efficacy, as evidenced by the PSEQ score increasing from 20 to 58. In particular, the integration of ADOC-H enabled specific, occupation-focused goal setting for activities involving the upper limb, which was highly appropriate for the clinical characteristics of a postoperative olecranon fracture. Once the patient achieved self-management of pain and anxiety, the focus shifted to behavioral change directed toward his established goals. Literature on chronic pain suggests that CBT-informed behavioral strategies following cognitive restructuring may improve disability and quality of life. Furthermore, setting target activities and tracking pain/activity levels using activity diaries in the early phase after total knee arthroplasty has been shown to improve pain management and increase activity levels [16]. In the present case, implementing pacing allowed for a gradual increase in activity without exacerbating pain from overactivity. Moreover, by integrating psychoeducation and functional training within the same OT session, the patient had immediate opportunities to correct the belief that "movement causes harm" through the direct experience of safe movement. This increased self-efficacy, the belief that "movement is safe", reduced fear-avoidance thinking related to painful joint movement. Consequently, this may have encouraged spontaneous use of the affected limb in daily activities, such as dressing, bathing, and PC operation. Increased opportunities for safe upper-limb use, together with natural postoperative recovery, pain reduction, and conventional rehabilitation, may have contributed to improvements in elbow function.

Limitations

As this is a single case report, the generalizability of the findings is limited. Furthermore, in the absence of a control group, we cannot definitively exclude the possibility that the observed improvements were influenced by natural clinical progression, pharmacological management, or interventions from other healthcare professionals. Additionally, since several outcome measures were self-reported, they may have been subject to subjective bias or the patient’s expectations of recovery. Future studies involving larger sample sizes, controlled designs, and various surgical procedures are warranted. Incorporating objective measures, such as physical activity tracking for long-term evaluation, would also provide more robust evidence of the efficacy of this approach.

## Conclusions

This case suggests that occupational therapy incorporating cognitive behavioral therapy-informed components (CBT-informed OT) is feasible after olecranon fracture surgery and may help address pain-related fear-avoidance during early rehabilitation. Over the 12-week intervention period, the patient showed clear improvement across multiple domains: pain on movement decreased (NRS 6 → 2), elbow active range of motion improved (−45/80 → −10/130), upper-limb disability decreased (QuickDASH 76.5 → 12.5), pain catastrophizing decreased (PCS 25 → 1), pain self-efficacy increased (PSEQ 20 → 58), and Canadian Occupational Performance Measure performance and satisfaction scores improved from 2 to 10 for both short-term (independence in dressing and bathing) and long-term (participation in work-related online meetings) occupational goals. A distinctive feature of this intervention was that meaningful occupations themselves served as the medium through which cognitive restructuring, graded goal setting, and pacing were operationalized, consistent with the clinical formulation that low pain self-efficacy and fear-avoidance, rather than catastrophizing alone, were the central psychological drivers in this case. These observed improvements should be interpreted cautiously, however, because natural postoperative recovery, concurrent analgesic use, and standard rehabilitation may also have contributed; no claim of treatment efficacy is made. The present findings are hypothesis-generating and warrant evaluation in controlled studies of patients with upper-limb fractures presenting with disproportionate pain-related fear-avoidance.
